# Conversational Interfaces for Health: Bibliometric Analysis of Grants, Publications, and Patents

**DOI:** 10.2196/14672

**Published:** 2019-11-18

**Authors:** Zhaopeng Xing, Fei Yu, Jian Du, Jennifer S Walker, Claire B Paulson, Nandita S Mani, Lixin Song

**Affiliations:** 1 Carolina Health Informatics Program University of North Carolina at Chapel Hill Chapel Hill, NC United States; 2 Health Science Library University of North Carolina at Chapel Hill Chapel Hill, NC United States; 3 National Institute of Health Data Science Peking University Beijing China; 4 School of Nursing University of North Carolina at Chapel Hill Chapel Hill, NC United States; 5 Lineberger Comprehensive Cancer Center University of North Carolina at Chapel Hill Chapel Hill, NC United States

**Keywords:** conversational interfaces, conversational agents, chatbots, artifical intelligence, healthcare, bibliometrics, social network, grants, publications, patents

## Abstract

**Background:**

Conversational interfaces (CIs) in different modalities have been developed for health purposes, such as health behavioral intervention, patient self-management, and clinical decision support. Despite growing research evidence supporting CIs’ potential, CI-related research is still in its infancy. There is a lack of systematic investigation that goes beyond publication review and presents the state of the art from perspectives of funding agencies, academia, and industry by incorporating CI-related public funding and patent activities.

**Objective:**

This study aimed to use data systematically extracted from multiple sources (ie, grant, publication, and patent databases) to investigate the development, research, and fund application of health-related CIs and associated stakeholders (ie, countries, organizations, and collaborators).

**Methods:**

A multifaceted search query was executed to retrieve records from 9 databases. Bibliometric analysis, social network analysis, and term co-occurrence analysis were conducted on the screened records.

**Results:**

This review included 42 funded projects, 428 research publications, and 162 patents. The total dollar amount of grants awarded was US $30,297,932, of which US $13,513,473 was awarded by US funding agencies and US $16,784,459 was funded by the Europe Commission. The top 3 funding agencies in the United States were the National Science Foundation, National Institutes of Health, and Agency for Healthcare Research and Quality. Boston Medical Center was awarded the largest combined grant size (US $2,246,437) for 4 projects. The authors of the publications were from 58 countries and 566 organizations; the top 3 most productive organizations were Northeastern University (United States), Universiti Teknologi MARA (Malaysia), and the French National Center for Scientific Research (CNRS; France). US researchers produced 114 publications. Although 82.0% (464/566) of the organizations engaged in interorganizational collaboration, 2 organizational research-collaboration clusters were observed with Northeastern University and CNRS as the central nodes. About 112 organizations from the United States and China filed 87.7% patents. IBM filed most patents (N=17). Only 5 patents were co-owned by different organizations, and there was no across-country collaboration on patenting activity. The terms *patient*, *child*, *elderly*, and *robot* were frequently discussed in the 3 record types. The terms related to mental and chronic issues were discussed mainly in grants and publications. The terms regarding multimodal interactions were widely mentioned as users’ communication modes with CIs in the identified records.

**Conclusions:**

Our findings provided an overview of the countries, organizations, and topic terms in funded projects, as well as the authorship, collaboration, content, and related information of research publications and patents. There is a lack of broad cross-sector partnerships among grant agencies, academia, and industry, particularly in the United States. Our results suggest a need to improve collaboration among public and private sectors and health care organizations in research and patent activities.

## Introduction

The emergence of conversational interfaces (CIs) enables users to talk to a machine [1]. Using conventional pattern match or natural language processing, CIs simulate human conversation through various interaction modalities [2-4]. For example, text-based CIs, also known as chatbots, are commonly presented on messaging platforms where users can converse with bots using textual input (eg, Facebook Messenger and Slack). Voice-based CIs incorporate a speech channel into the interface and are preferred over traditional graphical interfaces (eg, keyboard and screen) in visual or hands-off tasks [5]. Recent examples of voice-based CIs include intelligent personal assistants (eg, Apple Siri, Amazon Alexa, and Microsoft Cortana) and CIs with multimodalities such as embodied conversational agents (ECAs). Multimodality can be designed to engage verbal and nonverbal interactions (eg, gaze movement, facial expression, and gesture) between the CI system and users [6,7]. This mode can also be implemented using an embodied character and menu-based dialogue module to simulate a dialogue flow [8].

In health care, CIs have been adopted to provide complementary therapy and health behavioral interventions [9-11], assist patient self-management [12-15], and support clinical decision making [16-18]. With the growth of CIs for health, there have been increasing efforts in appraising research in CI design and applications. Laranjo et al [2] identified 17 studies that have investigated 14 unique CI applications for general health-related purposes. These studies reported that CIs have produced positive outcomes related to patient engagement and adherence and decreased self-reported symptoms. A review examined 40 studies regarding CI technologies in hospital settings and proposed a taxonomy that involved interaction context, dialogue types, and architecture attributes. Researchers reported that CIs were primarily designed for physician education and patient counseling purposes [3]. A review of 8 studies that applied CIs to treat mental illness [4] reported positive outcomes and user satisfaction yet inconsistent evaluation of CI technologies.

Despite the rapidly growing research evidence supporting the potentials of CI applications in health care, there is a lack of systematic investigation that goes beyond the published research and incorporates grant and patent activities. This will demonstrate the state of the art from the perspectives of funding agencies, academia, and industry. Research grants reflect funding agencies’ attention to and support for emerging research domains [19,20]. Publications and patents have been widely used to map the emergence of technologies and reveal research and development (R&D) activities [21,22]. A broad scope review using data from multiple sources, such as scientific publications, funding instruments, and patents, will thus provide an overview of the domain landscape and inform stakeholders [23,24] for better CI utilization and research. In addition, a recent report on artificial intelligence (AI) by World Intelligence Property Organization (WIPO) [25] identified the untapped opportunities that AI technologies have brought to health care and called for a broad collaboration and/or coordination among funding agencies, policymakers, researchers, and entrepreneurs. As one of the most promising health care applications of AI, CIs should be given sufficient attention. This study aimed to use data systematically extracted from multiple sources (ie, grant, publication, and patent databases) to investigate the development and research of health CIs. We examined the following 5 research questions:

- RQ1: Basic statistics of the collected records: how many grants, publications, and patents exist for CIs used for health purposes?
- RQ2: Analysis of grants: which funding agencies have granted the largest amount of funds in CIs and which organizations have received funds in CIs?
- RQ3: Analysis of research publications: who are the top contributors to research publications, who are their main collaborators, and how are they distributed geographically?
- RQ4: Analysis of patents: where were the patents filed (ie, country) and who were the most active patent assignees (ie, organizations)?
- RQ5: Analysis of topic terms: what terms were frequently addressed in grants, publications, and patents and what potential gaps can be identified for future research?

## Methods

### Data Collection

To identify grants, publications, and patents, we created a search strategy adapted from previously published works that include CI-related terms [1,2,21,26], such as *spoken dialogue system*, *conversational agent*, *chatbot*, *social robot*, *virtual agent*, *and question-answer system.* The health-related terms covered both generic health term variations and key stakeholders, such as *health*, *healthcare*, *medicine*, clinic, *physician*, *patient*, and *caregiver*. In addition, we used a snowball process to collect synonyms, term variations, and relevant terms to build a search term thesaurus for this study [27]. Search queries were modified for different databases (see Multimedia Appendix 1).

We systematically searched 9 databases to identify granted projects, research publications, and patents from January 2008 to December 2018 that were for health purposes (Table 1). Initial searches were conducted in August 2018 and updated in January 2019. The databases for grant search included Federal RePORTER and Community Research and Development Information Service (CORDIS). Federal RePORTER is a publicly available database of scientific funding projects by federal agencies, including National Institutes of Health (NIH), National Science Foundation (NSF), the Agency for Healthcare Research and Quality (AHRQ), Department of Agriculture, National Aeronautics and Space Administration, Department of Defense, Environmental Protection Agency, and Department of Education. For a broader coverage of funding records, we also searched the Europe Commission (EC) by using CORDIS, a primary information source of funded projects in the European Union countries.

The publication search was conducted in 6 bibliographic databases that index literature in technology, biomedical, and health sciences (Table 1). Publications were limited to conference papers, journal articles, and book sections and excluded meeting abstracts, editorials, letters, or lecture notes. We retrieved the filed and granted patents from Derwent Innovation Index (DII). Sponsored by Clarivate Analytics, DII is a widely used database in patent bibliometric analysis and has a broad coverage of over 37 million inventions and 70 million patents from 52 authorities in the world [24,28].

After searches were completed, the retrieved records from the 9 selected databases were aggregated and deduped in EndNote (Clarivate Analytics). Particularly, if a record had a duplication, we only kept the one which had complete data fields. We also cross-checked other data resources for the missing parts of the records.

**Table 1:** Databases of grants, publications, and patents.

Data type	Database searched
Grants	Federal RePORTER and Community Research and Development Information Service
Publications	Web of Science, PubMed, Scopus, EMBASE, Association for Computing Machinery Digital Library, and Cumulative Index of Nursing and Allied Health
Patents	Derwent Innovation Index

### Data Screening

**Figure 1:**
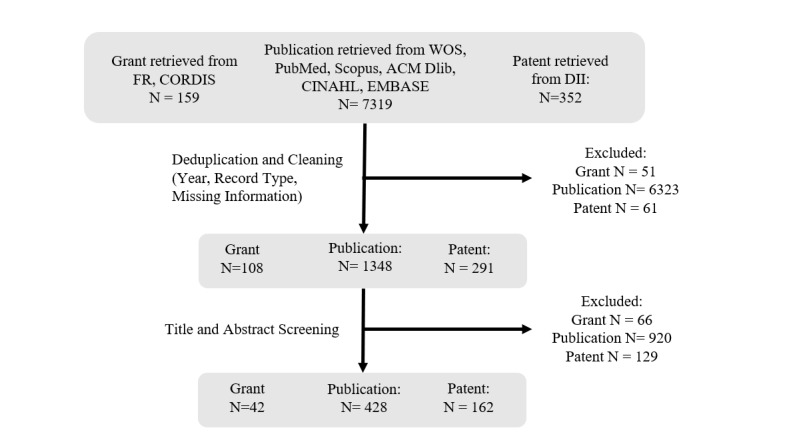
Data collection and screening.

We included grants, scientific publications, and patents that were relevant to the design, development, improvement, deployment, or evaluation of CIs for health purposes. A record was excluded if (1) it was awarded, published, or filed before 2008; (2) on cross-check, it missed any of the following fields: organization recipients of a grant, grant agency, grant size, or budget year; title, abstract, or author affiliation; and country, patent assignee, or priority date; and (3) it was irrelevant to CI technologies for health purposes. For example, we included the records that reported systems, programs, and interfaces, which enabled users to issue command requests or engage in dialogue/chats in different input modalities (eg, text based, spoken, menu selection, or multimodal). We excluded the records that did not involve or specify the abovementioned modes, such as pet robot, nonverbal virtual robots, and the Internet of Things. We also excluded the records that examined CIs for nonhealth purposes. For patents, we included the records that claimed CI inventions that can be used for health purposes. After deduping and cleaning the data, ZX manually screened the titles and abstracts of the records, and the records with uncertainty were discussed at team meetings and resolved by co-authors (Figure 1).

**Data Analysis**

We employed bibliometric analysis, social network analysis (SNA), and term co-occurrence analysis techniques to analyze the abstracted data (Table 2). Bibliometric analysis is the quantitative analysis of scientific publications [29]. It has been widely used for measuring research and patenting performance [21,30] and recently extended to measuring research funding dynamics [23,24]. SNA was originally developed for social structure study. Using bibliographic data, SNA expands the scope of bibliometrics by revealing the co-authorship among different research units [31,32]. Built on network theory and bibliometrics, the term co-occurrence analysis represents the knowledge components of a document by key terms and their co-occurrence relationship [33]. Researchers have used the term co-occurrence approach to explore potential research topics in scientific publications [34,35].

**Table 2:** Extracted data fields, analysis methods, and metrics.

Data sample	Data fields	Analysis methods	Metrics
Grant	Title, abstract, grant agency, granted organization, grant size, and grant start year	Bibliometric analysis, social network analysis, and term co-occurrence analysis	Number of funded projects, grant size, project counts, project duration, funding and recipient agency and organizations, and topic co-occurrence
Publication	Title, abstract, affiliation, and publication year	Bibliometric analysis, social network analysis, and term co-occurrence analysis	Number of publications, organizational network, and topic co-occurrence
Patent	Title, abstract, patent assignee, and the priority date^a^	Bibliometric analysis, social network analysis, and term co-occurrence analysis	Number of patents, organizational collaboration network, and topic co-occurrence

^a^We used the priority date as the time stamp for patent data, which is the date when the first patent application in a patent family was filed at a patent office (priority application) and which is often used to establish the priority of an invention [36].

We used both VOSviewer (version 1.6.9) and Tableau for data analysis and visualization. VOSviewer is a bibliometric network analysis software to conduct SNA for collaboration networks and term co-occurrence analysis for topic analysis [37,38]. For SNA, each organization was represented as a node, and each collaboration between 2 nodes was represented by a link. For the term co-occurrence analysis, key terms were extracted from the title and abstract of the records. We used a density map to visualize the disclosed key terms. The importance of a topic was measured by frequency of terms (ie, counts of a term) and its co-occurrence with other terms. The synonyms of extracted key terms were consolidated for visualization. For example, the extracted terms *child*, *children*, and *kid* were merged and standardized as *child*.

## Results

### The Number of Grants, Research Publications, and Patents

**Figure 2:**
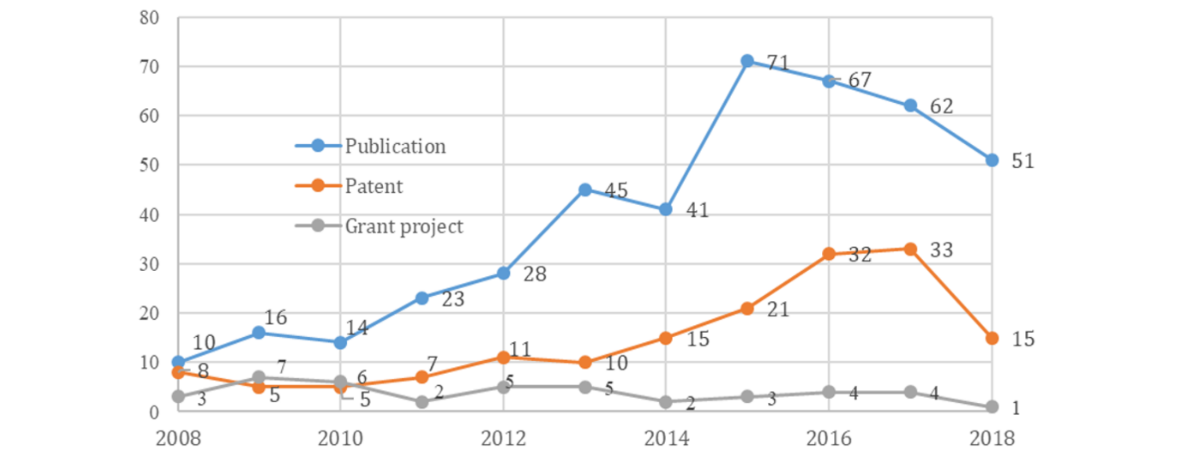
Number of grants, research publications, and patents between 2008 and 2018.

After data screening, we obtained a dataset with 42 grants, 428 research publications, and 162 patents related to CIs for health purposes. As displayed in Figure 2, the number of grants has remained steady from 2008 to 2018, with an average of 3.8 projects per year. The number of publications increased since 2010, with an average growth rate of 22.1% per year, which then reached a peak in 2015. The number of patents also grew from 2010 to 2017, with an annual growth rate of 22%. The US patent applications filed on and after 2018 are not publicly available until 18 months after the application's earliest filing date [36], causing the drop-in number of patent records in 2018.

### Analysis of Grants

**Figure 3:**
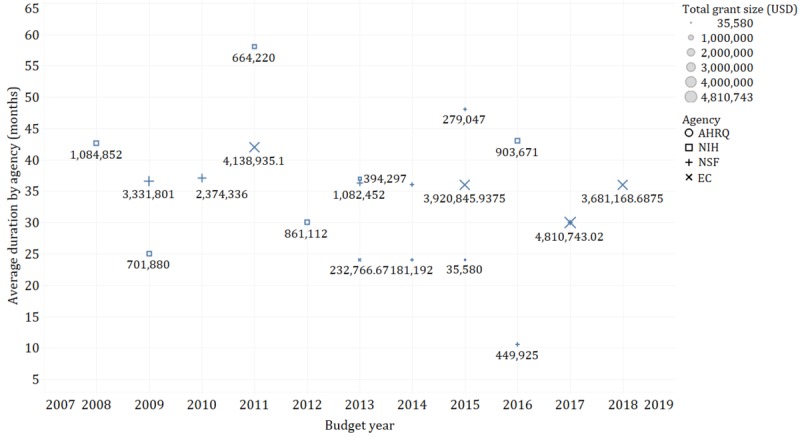
Grant size (USD) and project duration (months) from 2008 to 2018. AHRQ: Agency for Healthcare Research and Quality; EC: Europe Commission; NIH: National Institutes of Health; NSF: National Science Foundation.

Among the 42 funded research projects focusing on CI technologies for health purposes, the total dollar amount of grants awarded was US $30,297,932, of which US $13,513,473 was awarded by funding agencies in the United States, and US $16,784,459 was awarded by the EC (Table 3 and Figure 3). The top 3 funding agencies in the United States were NSF, NIH, and AHRQ, which overall funded 45.2% of the included projects. Among the 6 EC-funded projects identified in this study, the EC grants were allocated to 10 countries, that is, the United Kingdom, Spain, France, Italy, Germany, Norway, Belgium, Israel, Latvia, and Romania (Table 4), for either individual or collaborative projects.

**Table 3:** Grant count, size, and average duration of projects.

Agency	Project count	Percentage of total count	Grant size	Percentage of total grants	Months of project, mean (SD)
National Science Foundation (the United States)	19	45.2	US $8,686,669	28.67	33.9 (15.9)
National Institutes of Health (the United States)	16	38.1	US $4,645,612	15.33	34.9 (17.4)
Agency for Healthcare Research and Quality (the United States)	1	2.4	US $181,192	0.60	24.0 (N/A^a^)
Europe Commission (European Union)	6	14.3	US $16,784,459	55.40	33.0 (6.7)

^a^Not applicable.

**Table 4:** The grant size and number of grant recipients by country.

Country	Organization recipients count	Total grant size
United States	27	US $13,513,473
United Kingdom	9	US $3,914,721
Spain	8	US $3,048,271
France	5	US $2,350,823
Italy	5	US $2,567,397
Germany	4	US $2,120,652
Norway	2	US $1,392,323
Belgium	1	US $330,315
Israel	1	US $218,738
Latvia	1	US $639,540
Romania	1	US $201,680

**Figure 4:**
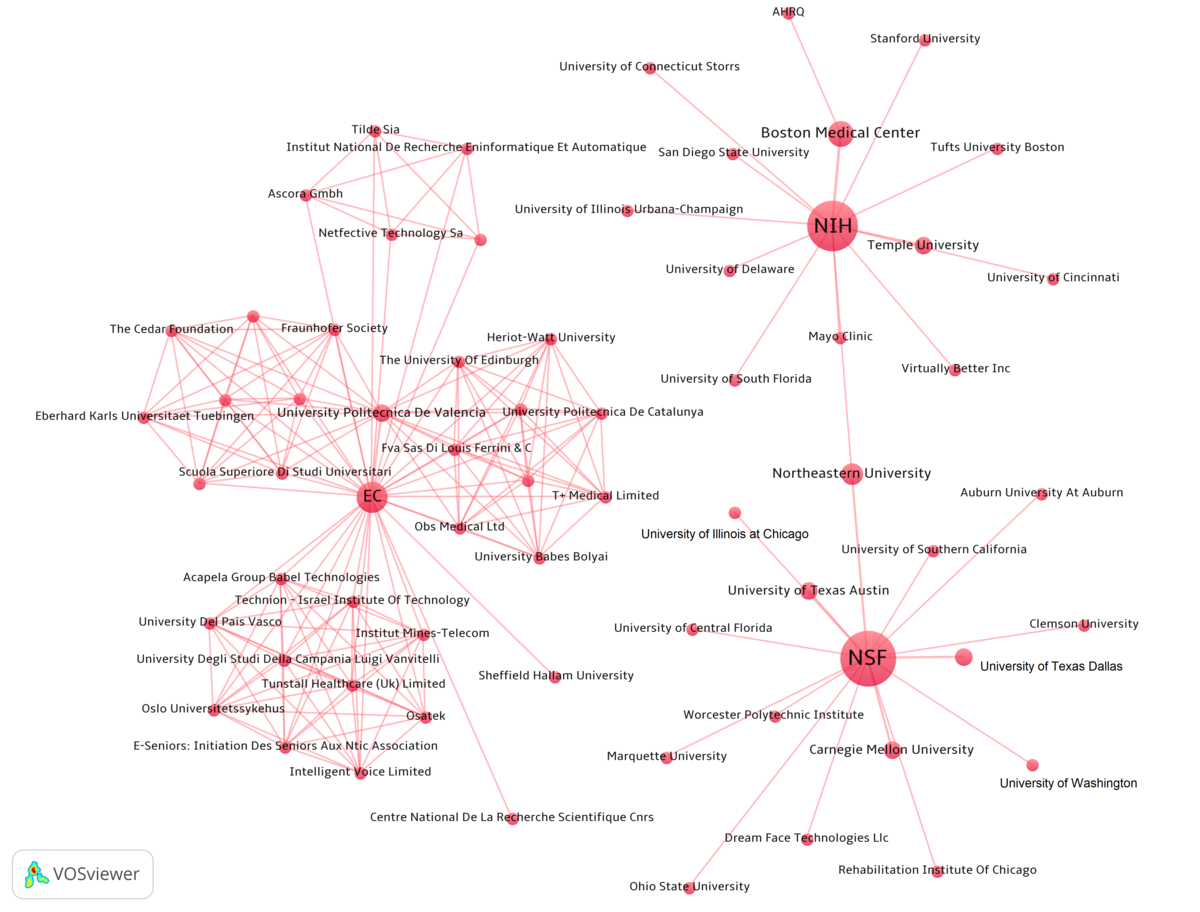
Grant agencies and granted organizations network. AHRQ: Agency for Healthcare Research and Quality; EC: Europe Commission; NIH: National Institutes of Health; NSF: National Science Foundation.

The 42 funded projects involve a total of 64 granted organizations (Figure 4). Among the 27 US grantee organizations, 26 were research institutes. Boston Medical Center was awarded the largest grant size with 3 projects funded by NIH with a total amount of US $2,065,245 and 1 project funded by AHRQ with the amount of US $181,192. These 4 projects were granted for designing and evaluating CIs to improve patients’ engagement [39], for palliative care of patients with advanced illness, for reducing cardiopulmonary rehospitalization [40], and for treating comorbid depression (no outcomes reported). In Europe, the EC funded 18 private companies and 19 academic institutes. The University of Edinburg received the largest grant for a project that applied CI technology to support the treatment of major depression (ie, US $1,758,786) [41].

The project duration varied from 3 to 69 months. For example, Auburn University and the University of South Florida were granted 3-month funding from NSF and NIH, respectively. The University of Delaware received funding that lasted for 69 months.

### Analysis of Research Publications

This study identified a total of 428 publications authored by researchers from 58 countries. As shown in Table 5, the researchers from the United States published 114 manuscripts on CI technologies for health purposes. The other countries that have published the most about this line of work included Japan (n_publication_=34), France (n_publication_ =33), China (n_publication_=28), the United Kingdom (n_publication_=24), Italy (n_publication_=21), Malaysia (n_publication_=20), Germany (n_publication_=17), and the Netherlands (n_publication_=16). Overall, researchers in European countries produced about 31.1% of the total publications compared with 19.2% by researchers in Asian countries.

Among the 566 organizations with which the authors were affiliated, 95 organizations (16.8%) contributed at least two publications, including 85 academic institutions, 6 hospitals, and 4 companies or private organizations. As shown in Table 5, the top 3 most productive institutes were Northeastern University, the Universiti Teknologi MARA System in Malaysia, and the French National Center for Scientific Research (also known as and shown as CNRS).

**Figure 5:**
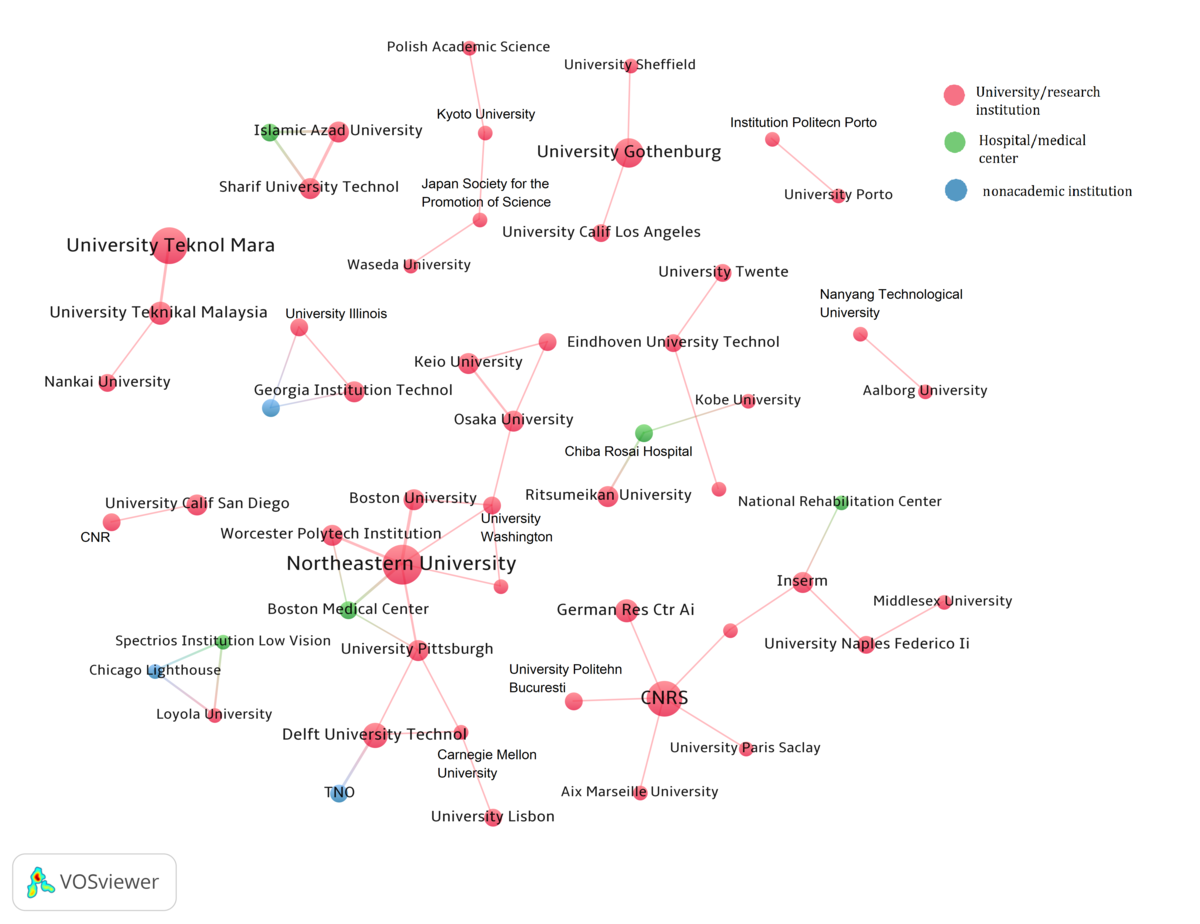
Publication organizational co-authorship network at country level. Each node represents an organization, and the size of the node represents publication count. CNRS: National Center for Scientific Research.

Regarding organizational collaboration, 246 of 428 publications (57.5%) were co-authored by researchers from at least two organizations. Overall, 463 organizations participated in interorganizational collaborations, whereas 303 organizations had more than 1 external collaborator. The top 5 institutes were CNRS (20 collaborators), Inserm (14 collaborators), Osaka University (13 collaborators), Northeastern University (11 collaborators), and the University of Naples Federico II (11 collaborators; Figure 5). A total of 2 collaboration clusters were identified from the organizational collaboration network with Northeastern University and CNRS as the central nodes.

**Table 5:** The number of publications by country and organization (Top 10), N=248.

Rank	Publications by country		Publication by organization	
	Country	Publication count, n (%)	Organization	Publication count, n (%)
1	United States	114 (26.6)	Northeastern University (US)	15 (3.5)
2	Japan	34 (7.9)	University Teknologi MARA (Malaysia)	13 (3.0)
3	France	33 (7.7)	CNRS (France)	12 (2.8)
4	China	28 (6.5)	University of South California (US)	9 (2.1)
5	United Kingdom	24 (5.1)	University of Gothenburg (Sweden)	8 (1.9)
6	Italy	21 (4.9)	Delft University of Technology (Netherlands)	6 (1.4)
7	Spain	21 (4.9)	Istanbul Technical University (Turkey)	6 (1.4)
8	Malaysia	20 (4.7)	University Carlos III de Madrid (Spain)	5 (1.2)
9	Germany	17 (4.0)	Universiti Teknikal Malaysia Melaka (Malaysia)	5 (1.2)
10	Nether-lands	16 (3.7)	German Research Center for Artificial Intelligence (Germany)	5 (1.2)

**Figure 6:**
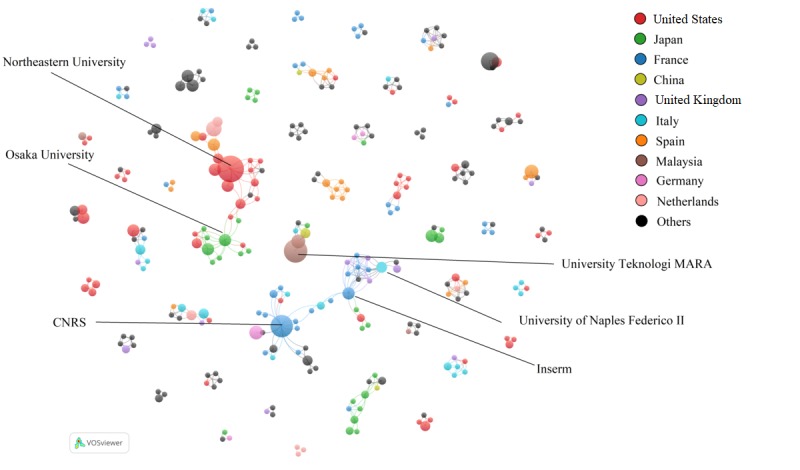
Publication co-authorship network at the organizational level. The organizations shown are those with more than 1 publication.

In addition to academic institutions, 5 hospitals/medical centers (ie, Chiba Rosal Hospital, Mahak Hospital, Boston Medical Center, Spectrios Institute for Low Vision, and National Rehabilitation Center for Persons with Disabilities in Japan), and 3 nonacademic institutions (ie, TNO, IBM, and Chicago Lighthouse) were involved in the collaboration network. The only collaboration across 3 types of organizations was among Loyola University, Chicago Lighthouse (a nonprofit social service organization), and the Spectrios Institute for Low Vision (Figure 6).

### Analysis of Patents

Among the 162 patents reviewed in this study, the United States and China filed the majority of the patent inventions, 78 and 69 respectively, and they are followed by Japan (n_patent_ =7) and South Korea (n_patent_ =6). Regarding the patent assignees, 112 organizations were identified, including 98 private companies and 14 research institutes. Among research institutes, 9 were based in China, 2 in Japan, 2 in the United States, and 1 in South Korea. IBM filed more patents (n_patent_ =17) than all the other organizations or companies. Organizations that filed more than 2 patents were Vocollect Healthcare (n_patent_ =6), Google (n_patent_ =4), Next It Corp (n_patent_ =3) and Samsung (n_patent_ =3).

With regard to the collaboration among patent assignees, we only identified 5 collaboration patents that were co-assigned to 5 pairs of organizations in the United States, Japan, and China. They included (1) Puretech Management Inc and Bose Corp (United States), (2) Next It Corp and Verint Americas Inc (United States), (3) University of Kyoto and Toyota (Japan), (4) Jiangsu province Hospital and Nanjing Zhongyue Information Technology Company (China), and (5) University of South China Technology and Guanzhou Lvsong Biotechnology Company (China). Furthermore, this study did not find any cross-country patent collaborations among identified assignees.

### Analysis of Topic Terms

#### Grants

**Figure 7:**
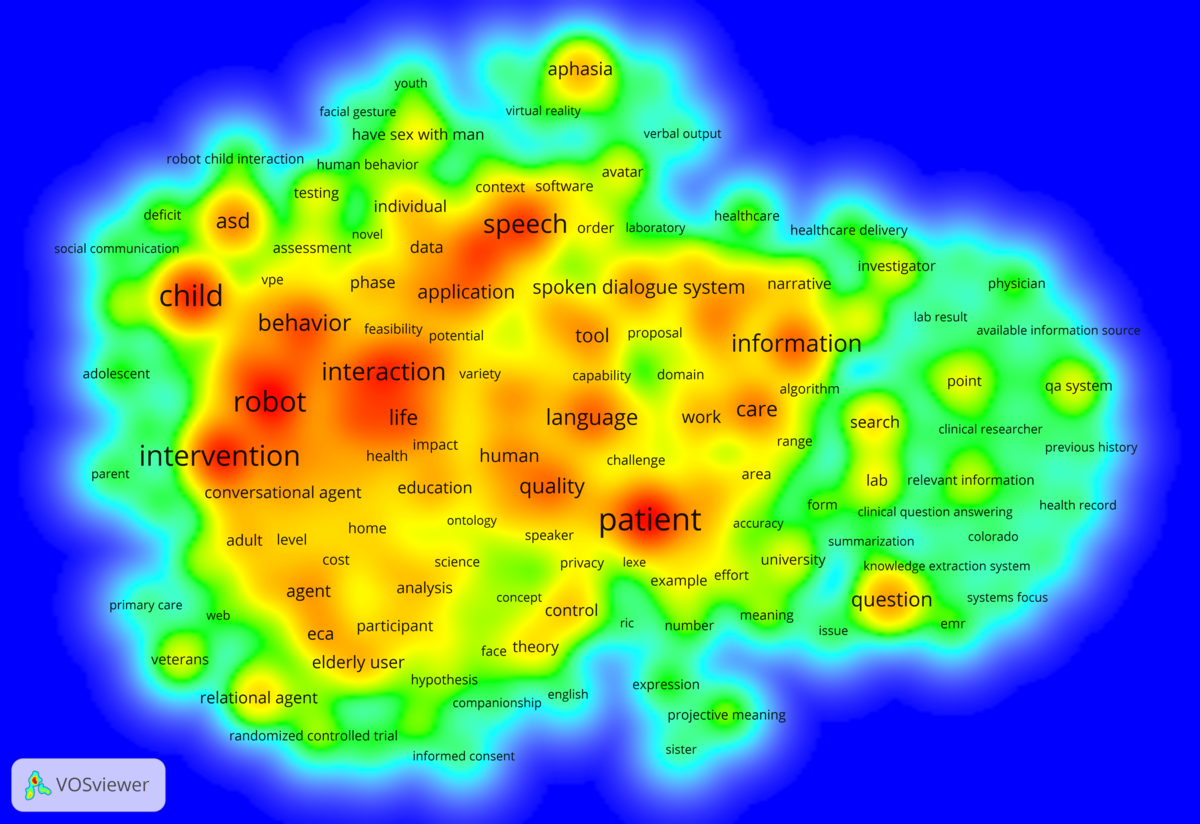
Heatmap of the topic terms that occurred in more than 4 granted projects.

The most commonly occurring terms (Figure 7) included *patient*, *child*, *intervention*, *robot*, and speech. The grants primarily focused on the *patient*, and 7 projects targeted the *child* or *elderly* populations. In addition, the term *asd* (ie, autism spectrum disorder [ASD]) was in very close proximity with *child* and occurred in 5 funded projects. For example, an NIH-sponsored project at the University of Connecticut and the University of Delaware in 2009 used social robots to support children with ASD. The term *elderly* was also addressed in 7 grants. For example, Auburn University and Clemson University proposed a project, funded by NSF in 2009, aimed to use CI to improve older adults’ quality of life. Furthermore, *speech* seems to be a preferred interaction modality in the proposals. For example, the University of Texas Dallas was funded by NSF to create a speech-enabled CI, which aimed to help individuals with hearing impairment and autistic children improve communication skills.

#### Publications

**Figure 8:**
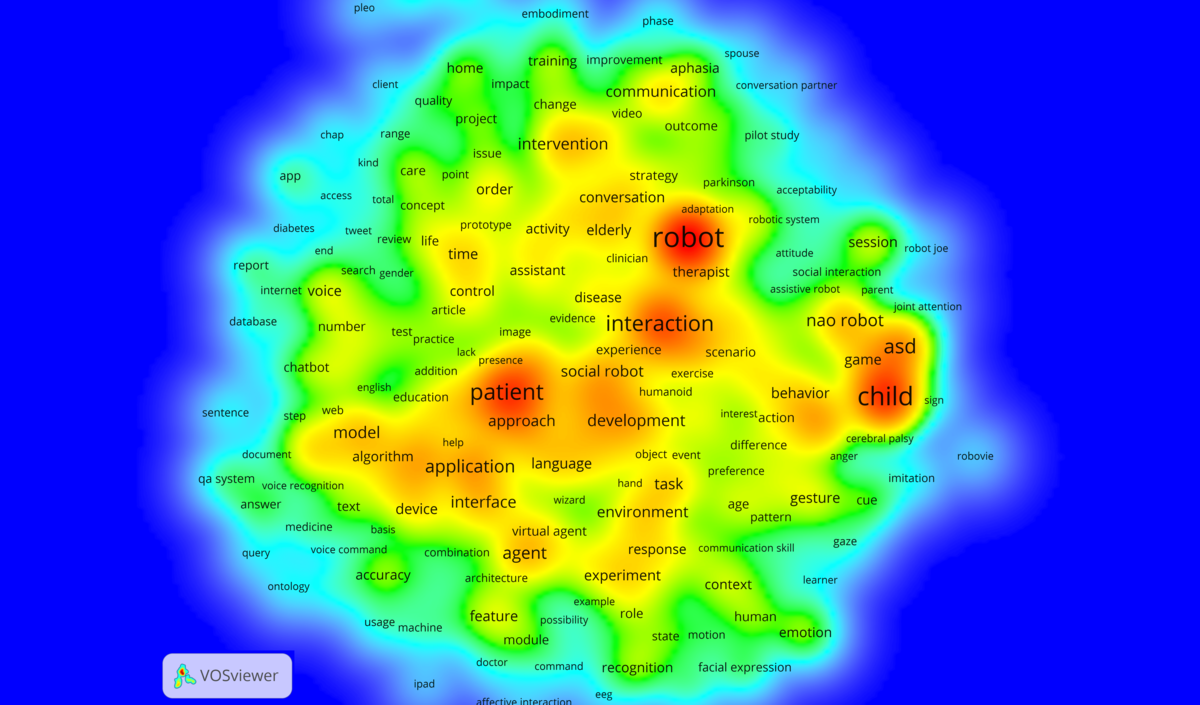
Heatmap of the topic terms that occurred in more than 9 publications.

The terms with top occurrence in publications were *robot*, *patient*, and *child* (Figure 8). In addition, *asd* appeared together with *child* in 46 publications [42,43]. Other disease-related terms discussed in the publications included *aphasia* (3.7%, 16/428) [44,45], *cerebral palsy* (2.8%, 12/428) [46,47], *stroke* (2.6%, 11/428) [48,49], *cancer* (1.4%, 6/428) [50,51]. The key term *elderly* was addressed in 42 publications [52,53]. The term *multimodal* occurred in 39 publications [54,55], and specific terms associated with multimodal interaction were *gesture* (10.3%, 44/428), *voice* (10.0%, 43/428), and *facial expression* (5.1%, 22/428). Furthermore, *NAO robot*, a humanoid robot developed by SoftBank Robotics, was investigated in 87 publications (20.3%) [42,43].

#### Patents

**Figure 9:**
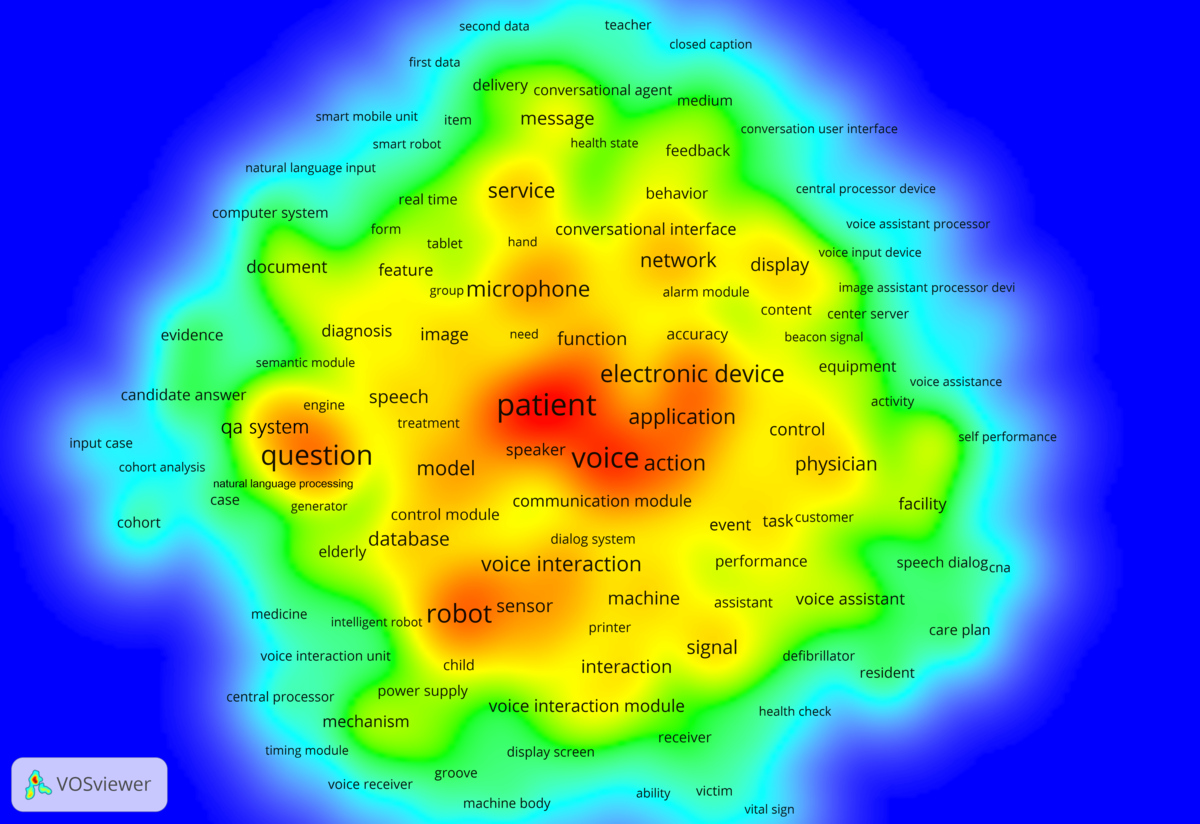
Heatmap of the topic terms that occurred in more than 7 patents.

The terms with top occurrence in patents (Figure 9) included *patient*, *voice*, and *electronic device*. Other key terms reflected the system components of CIs [56,57], such as *speaker* (18.5%, 30/162), *microphone* (15.4%, 25/162), and *communication module* (6.2%, 10/162). In addition, the terms *robot* (n_patent_=34, 21.0%, 34/162), *sensor* (14.8%, 24/162), and *voice interaction* (42.6%, 69/162) were closely positioned and co-occurred frequently [58,59]. Another frequently occurred term was *question* (16%, 26/162), which also appeared closely with question answering systems, (9.3%, 15/162) [60,61]. The stakeholders addressed in the patents included not only *patient* but also *physician* (3.1%, 5/162) and *elderly* (2.5%, 4/162) [62,63].

## Discussion

### Principal Findings

This is the first study to systematically examine emerging CI technologies for health purposes using grants, research publications, and patent data. We found increasing efforts in recent years in exploring health-related applications of CI.

There has been an increase in government funding support in this field in the United States and European countries since 2008. The total dollar amount of EC grants was higher than that of US grants. Research institutes and private organizations have been involved and awarded almost equally in European countries, whereas research institutions were the only type of awardee in the United States. These results may suggest that funding agencies in the United States need to encourage more collaboration between academia and industry.

The research publications are mainly from a few countries and institutions, and there is a lack of international collaborations across countries. US researchers have been leading in the number of publications about CIs for health. Northeastern University was the leading institute that has focused on the design and evaluation of CIs for health education and behavioral intervention in clinical settings, such as information access to clinical trials for cancer [39], depression therapy [64], or spinal cord injury recovery [65]. This university has established a wide research collaboration network, but its collaborators are mainly domestic institutions. Researchers in European countries produced about one-third of the included publications. CNRS, a prominent French research institute, has applied CIs to tackle health issues such as substance use disorder [66] and depression [67]. The collaboration in European countries is centered on France, and collaboration with US institutions is rarely observed, suggesting that CI research is still geographically isolated, and more cross-country collaboration could be encouraged. The Universiti Teknologi MARA system in Malaysia was the major contributor to research publications in Asia. Their research has focused on adopting the robot *NAO* in interventions and rehabilitation for patients with mental or brain disorders (eg, ASD [68] and cerebral palsy [69]). Osaka University in Japan had the most research collaborators compared with other Asian institutes. The institutions in Malaysia and China, on the other hand, were not actively involved in CI research collaboration. In addition to the lack of collaboration between countries, there is also a lack of collaboration between researchers and health care professionals. Several hospitals and medical centers engaged in interorganizational collaborations, but the number of such collaborations is still small. This may explain the existing challenge of insufficient clinical evaluation of CIs, inconsistent results, and high concern for patient safety, which were disclosed by previous studies [2,70,71]. In addition, the function of CIs in patient care requires substantial domain knowledge and an adequate understanding of human emotion [72]. Given these facts, the involvement of health care providers is important in design and clinical evaluation for better user-centered CI functions and improved patient outcomes.

The United States and China were the 2 most active countries in patenting activities, but the major players were different. In the United States, private companies (eg, IBM, Vocollect Healthcare, and Google) have dominated patent applications, whereas in China, academic institutions are most actively engaged in patent applications. This finding is consistent with results from the WIPO’s report on AI [25], which suggested that China has paid increasing attention to research-innovation translation. In addition, co-owned patents among university and companies were rarely identified in this study, which may suggest that academia-industry collaboration in patent activities related to CIs for health is currently uncommon, and further efforts are required to bridge this gap.

This study also presented key topic terms regarding the targeted users, health issues, and interaction modality. Overall, both *patient* and *robot* were addressed by all 3 types of records. *Child* and *elderly* appear to be 2 major user-related terms frequently discussed. For example, Northeastern University investigated computer-based ECA, which served as a humanoid assistant for patients with low literacy [73,74]. IBM's inventions, which primarily concerned clinical QA systems, helped physicians and other medical professionals to search medication evidence and supported their clinical decisions [60,61].

In terms of targeted health issues, this study found CIs were mainly used for addressing mental health and chronic diseases. The *asd* is the most frequently investigated term in grant applications and publications. It often occurred along with *children* and *robot*. This finding is consistent with the results from previous studies [2,75,76] that robots have been widely used to treat children with ASD by promoting their social behaviors and improving their communication skills. Our results also revealed that CIs were adopted to manage chronic diseases, such as stroke [46], cancer [47], and dementia [77]. These health issues usually require long-term treatment and intensive care. CIs, as a supplement to health care providers, can be used to promote communication and provide a support companion for patients with ASD [42,43] and to deliver interventions and management for patients with chronic diseases [48-51].

Regarding the human-CI interaction modality, we found that the CI interface was favorably presented as *robot* and that multimodality was widely discussed in publications and patents. For example, NAO robots interacted with users in both verbal and nonverbal modes and provided face-to-face communication and physical touch. More than 80 publications reported findings about the applications of this humanoid robot among patients with various health issues, including improving communication skills for children with ASD (eg, [42,43]), reducing distress for cancer patients (eg, [50]), and providing functional and emotional support for the elderly (eg, [52,78]). NAO’s hybrid communication channels (eg, speech, eye contact, and body movement) contributed to users’ in-depth engagement to achieve desired behavior change. This may suggest that the multimodal robot has become one of the main forms of CIs for health purposes. However, it does not mean that this is the only or best way to design or implement CIs for health. Instead, different approaches in presenting CIs for health can potentially benefit health care resolutions in different clinical scenarios [2,3]. Overall, empirical evidence has confirmed the positive effects of CIs’ multimodal interface on medication adherence, social activity, and elderly patients’ learning processes [3]. Nevertheless, there is a lack of consistency and evidence of long-term clinical effects of the CIs [75]. Therefore, future research should deepen the understanding of user interaction with multimodal CI agents and the corresponding impacts on patient outcomes.

### Limitations

The study has several limitations. First, the selection of databases in this study introduced bias because we included only records released in English. Therefore, the results did not reflect any grants, research, and patenting activities reported in other languages. Second, during the data screening process, although the uncertain cases were reviewed and discussed by co-authors, only the first author screened the entire retrieved records, which may have led to bias in records inclusion and exclusion and consequently affected the results. Third, privately funded projects were not included in the analysis of grants data because of lack of access to private funding databases. Finally, the 3 types of data (ie, grants, publications, and patents) were retrieved and analyzed independently. There were no direct data mapping and integration across these data sources. Thus, results in this study should not be interpreted with respect to the relationship between public research investment, research outcomes, and patenting activities.

### Conclusions

This study systematically analyzed CI technologies for health purposes using data extracted from multiple information sources. Our findings provided an overview of the countries, organizations, and topic terms in funding activities as well as the authorship, collaboration, contents, and related information of research publications and patents. Overall, the inclusion of grants and patents in addition to publications has presented complementary insights into the R&D landscape of the use of CIs for health purposes. Our results have shown that there is a lack of cross-sector collaboration among grantees in the United States. In addition, international collaboration in research should be encouraged among the institutes to sustain the growth of CI application in health. The academic-industrial collaboration should also be fostered in patenting activities. Although current CIs have focused extensively on mental health problems and chronic diseases, future research needs to extend CI technologies to more diverse health issues via safer and more engaging multimodal interfaces.

## Appendix

Multimedia Appendix 1 Search query.

## Abbreviations

AHRQ: Agency for Healthcare Research and Quality
ASD: autism spectrum disorder
CIs: conversational interfaces
CNRS: National Center for Scientific Research
CORDIS: Community Research and Development Information Service
DII: Derwent Innovation Index
EC: Europe Commission
EU: European Union
NIH: National Institutes of Health
NSF: National Science Foundation
PI: principal investigator
R&D: research and development
WIPO: World Intelligence Property Organization
