# Supplementary material for: Conversational Interfaces for Health: Bibliometric Analysis of Grants, Publications, and Patents
Source: J Med Internet Res. 2019 Nov 18;21(11):e14672. doi: 10.2196/14672 (PMC6887814; doi:10.2196/14672)
Supplement: Multimedia Appendix 1 [file jmir_v21i11e14672_app1.docx]

**Search**: Using Conversational interfaces in Healthcare settings

**Publication Databases**: PubMed, Embase, Web of Science, Scopus, CINAHL, ACM DL

**Patent database**: Web of Science Derwent Innovation Index

**Grant databases**: Federal RePORTER

**Time Frame**: 2008-2018

**Search query:**

**1. Conversational interfaces:**

TI=("chat bot" OR "automated dialog system" OR chatbot OR chatterbot OR "conversation agent" OR "conversation assistance" OR "conversation assistant" OR "conversation assistive" OR "conversation avatar" OR "conversation robot" OR "conversational agent" OR "conversational assistance" OR "conversational assistant" OR "conversational assistive" OR "conversational avatar" OR "conversational bot" OR "conversational interaction" OR "conversational interface" OR "conversational robot" OR "conversational use interface" OR "embodied conversational agent" OR "embodied conversational avatar" OR "humanoid agent" OR "humanoid assistant" OR "humanoid avatar" OR "humanoid interaction" OR "humanoid robot" OR "interactive voice interface" OR "question answer system" OR "question answering system" OR "relational agent" OR "relational assistance" OR "relational assistant" OR "relational assistive" OR "relational avatar" OR "relational robot" OR "smart speaker" OR "social agent" OR "social assistant" OR "social avatar" OR "social robot" OR "speech activated agent" OR "speech activated assistant" OR "speech activated interaction" OR "speech activated robot" OR "speech activated system" OR "speech controlled robot" OR "speech enabled agent" OR "speech enabled assistant" OR "speech enabled interaction" OR "speech enabled robot" OR "speech enabled system" OR "speech generated interaction" OR "spoken dialogue agent" OR "spoken dialogue assistant" OR "spoken dialogue system" OR "talking avatar" OR "talking robot" OR "utterance avatar" OR "utterance robot" OR "virtual agent" OR "virtual assistance" OR "virtual assistant" OR "virtual assistive" OR "virtual human interaction" OR "voice activated agent" OR "voice activated assist" OR "voice activated assistance" OR "voice activated assistant" OR "voice activated assistive" OR "voice activated interaction" OR "voice activated system" OR "voice activated system" OR "voice agent" OR "voice assist" OR "voice assistance" OR "voice assistant" OR "voice assistive" OR "voice controlled agent" OR "voice controlled assist" OR "voice controlled assistance" OR "voice controlled assistant" OR "voice controlled assistive" OR "voice controlled robot" OR "voice controlled system" OR "voice driven agent" OR "voice driven assist" OR "voice driven assistance" OR "voice driven assistant" OR "voice driven assistive" OR "voice driven interaction" OR "voice driven interface" OR "voice driven robot" OR "voice driven system" OR "voice enabled agent" OR "voice enabled assist" OR "voice enabled assistance" OR "voice enabled assistant" OR "voice enabled assistive" OR "voice enabled interaction" OR "voice enabled robot" OR "voice enabled system" OR "voice generated agent" OR "voice generated assist" OR "voice generated assistance" OR "voice generated assistant" OR "voice generated assistive" OR "voice generated interaction" OR "voice generated interface" OR "voice generated robot" OR "voice generated system" OR "voice interaction" OR "voice robot" OR "embodied conversational assistant" OR "multimodal interaction" OR "multimodal interface")

**2. Health-related terms:**

(care OR caregiver OR "caregivers clinic" OR clinics OR clinical OR clinician OR clinicians OR doctor OR doctors OR disease OR diseases OR fitness OR health OR healthcare OR hospital OR hospitals OR illness OR illnesses OR Self-management OR medical OR medicine OR nurse OR nurses OR nursing OR patient OR patients OR physician OR physicians OR symptom OR symptoms OR treatment OR disorder OR disorders OR disability OR disabilities OR disabled OR sickness OR sick OR prevention OR therap* OR diagnos* OR rehabilat* OR clinic* OR homecare OR telemedicine OR ailment)
